# Recent Applications of Electricity

**Published:** 1902-11-15

**Authors:** George Parker

**Affiliations:** Physician Bristol General Hospital


### Hospital Clinics and Medical Progress.

### RECENT APPLICATIONS OF ELECTRICITY.

By George Parker, M.A., M.D.Camb., Physician Bristol General Hospital.

### II. Current from the Main.—High Frequency Currents.

In the last paper some methods were indicated by which the alternating or sinusoidal current from the main could be used for electric baths under complete control and without the risk of earth currents. For other details we may refer to an article on page 77. With due care then this current from the main may very largely replace the troublesome primary batteries for therapeutic treatment. It can be applied by arm or leg baths, wet sponges, full length baths, and, in some cases, by large, well-moistened electrodes. When ordinary electrodes are used the sensation is unpleasant, but when applied through water the current is far more agreeable than those from hand batteries. Moreover its power of influencing nutrition has been found to be as good as that of a galvanic current, and for protracted treatment, as in infantile paralysis, it is unequalled, especially if in addition to the air bath we occasionally pick out specially degenerated muscles by a sponge electrode. It would be an advantage if we possessed a ready means of measuring the intensity of this current, but instruments for this purpose have hitherto been unsatisfactory.

If we have a continuous current from the main the patient should never be in series with the rheostat, but always in "shunt" circuit, and a reliable "cut out" should be employed. Hedley and other authorities lay down that lamps and fuses are not trustworthy in working with continuous currents from the main. Since we cannot produce directly from them a secondary current as we can with alternating mains, perhaps the plan most free from risk is to drive a small motor dynamo from the main current, and to obtain from it a small continuous current. A fairly good method with a double shunt is that recommended by Jacoby.[2] He advises that (1) a wire resistance with sufficient heat-radiating surface should be fixed at the point of entrance of the main current which will limit the intake to one ampere. (2) A second resistance should be placed below this from which the current should be shunted. (3) This shunt current should be passed through another controller from which a final branch is derived for application to the patient. However, unless we use a motor dynamo the patient must be insulated, or both wires must be controlled by sufficient resistances.

When the electric current is employed for improving the nutrition of wasted muscles, as in the case of a paralysed limb, and not for diagnostic purposes, it is found, as we have seen, that the steady application of a current for a given time is much less valuable than when it is broken up by periods of rest. The nerve muscle machine in the human body is so constructed that contraction is followed by relaxation, katabolism by anabolism, expenditure of energy by fresh storage and accumulation. The growth of a muscle depends on an adequate combination of the two. Frequent periods of exercise, each followed by a period of rest, are necessary for its development. The heart muscle may contract 60 times a minute, but the greater part of the cycle is devoted to rest. Hence it is not surprising that Debedat's experiments have shown that the use of currents which are cut off about 30 times a minute has much greater effect in causing growth of a wasted muscle than if we use the same current without stopping. The interruptions of a Faradic current are far too short and frequent to enable the muscle to feed in the intervals, and it is therefore desirable, whatever current is used, to insert a Metronome to produce these stops every two seconds. An ordinary instrument, such as is employed by musicians, can be easily adapted for the purpose, and it becomes a question whether the pauses might not with advantage be made longer than the period of flow. Possibly the best results might be obtained by imitating the systole and relaxation of the cardiac cycle.

Among the therapeutic uses of the sinusoidal current from the main, we find that when the full length bath is employed, a remarkable tonic effect is produced; thus children suffering from rickets or strumous conditions often show speedy improvement. Chlorosis and spinal neurasthenia are rapidly affected, and in muscular rheumatism and subacute and chronic articular forms great relief can be obtained. In the same way the results of an attack of gout, the tenderness and swelling may be quickly removed. Hedley[3] refers also to the remarkable success obtained in acne and chronic eczema which have disappeared in a number of cases under this treatment.

As a means of applying electricity without pain, and in a thorough manner the sinusoidal current bath, either local or general, is largely superseding the older methods. Thus it is a convenient and potent method for treating peripheral paralyses, whether from lead alcohol or injuries, infantile paralysis, and for checking the advance of progressive muscular atrophies. In diseases due to spinal or cerebral lesions, we are confronted with the question how far the seat of the lesion can be influenced by electricity? We have not the means of giving a definite reply with the same certainly that we can as to peripheral lesions, but in a hemiplegia from cerebral hæmorrhage, for instance, the recovery of muscular power is rendered much quicker if a short course of electrical treatment is given a month or more after the attack. Moreover the noticeable improvement in general health and the increase of metabolism and excretion, which general baths cause indirectly aids recovery. When we wish to influence especially one part of the body a movable electrode, shaped like a paddle or tennis racket is held close to it. Thus a rheumatic joint is best treated by such an electrode or one formed by a large wet sponge held on the joint while the patient is lying in the bath, the other electrode conveying the current to the whole mass of water in the bath. Salt should never be added to the water of baths in which two electrodes are immersed, since, by rendering the water a better conductor, it causes a large amount of current to pass by and outside the patient.

It may be asked, how does the sinusoidal current differ from the Faradic? In both we have an alternating current, but in the former the changes in direction and magnitude are made gradually and the effect is less painful than in the sudden rises and falls of Faradism. We get just as much muscular stimulation with less pain, and it is "the current par excellence for improvement of muscular nutrition when this is impaired by a failure of proper stimulation."[4] Where Faradism cannot produce muscular contractions, where the muscles are wasted, or where subacute neuralgia is present this current gives excellent results. If, however, sensory effects and cutaneous stimulation are needed then the sharp action of the Faradic current is desirable. The current from the main is not, perhaps, a perfectly sinusoidal one, but it may be regarded as one for all practical purposes. Where no public currents or only a continuous one can be obtained a true sinusoidal current can be generated from a special machine, and here the rate of alternation can be regulated as we wish.

### High Frequency Currents.

The use of these currents is due chiefly to the discoveries of Tesla and d'Arsonval, and their application forms one of the most interesting chapters of recent electrical work. Their physiological effects are limited chiefly to metabolic changes. They cause no muscular contractions and produce no sensations except that of slight warmth, though some analgesia and failure to respond to motor stimuli follows their application for a short time. The blood pressure is first lowered and afterwards remains higher than normal. The absorption of Oxygen and the production of $CO_2$ may be doubled, and the output of urea and the action of the kidneys, lungs and skin is increased. Thus these currents are employed in rheumatism, gout, diabetes, various skin diseases, and for the relief of pain. One curious and successful application is the painless treatment of piles by a rectal electrode connected with one pole of a high frequency apparatus. From their physiological properties it is not surprising that they have been found useful in or after diseases attended by defective nutrition, and here they seem to produce better assimilation with increased appetite, energy and a return of sleep. They have, too, been used with success in sciatica and neuritis or lumbago. Lewis Jones[5] thinks that their use is merely a method of general electrification and that the results are similar to those obtained by static machines or the electric bath, though they have certain advantages for local applications, as in the treatment of skin diseases. Crombie and Bokenham[6] have recently treated 17 cases of dilatation of the stomach with brilliant success by these currents. The outline of the stomach invariably decreased from half to three-quarters of an inch after each application. This result could be always shown by marking out the area as given by gentle percussion, and repeating the process after the treatment. In from 10 to 20 sittings the stomach regained its normal size and motility. The action seemed to be confined to an improvement of the muscular apparatus, and no direct effect on the glands was noticed. Careful diet was employed, but some of the patients had failed to improve under dietetic and other treatment for years or months before the electric treatment was added.

The currents may be obtained by an alternating current from the main, which is raised in a Tesla transformer to several thousand volts, and then passed through a condenser. More commonly the source is a Wimshurst machine or Ruhmkorff spark coil fitted with a good interrupter and giving a spark 10 inches or more in length. From this the current passes to the inside of two Leyden jars with an adjustable spark-gap. Thirdly from the outside of these jars the high frequency currents, which are applied to the patient, are derived. In this new circuit a small helix or solenoid of thick copper is inserted which can be tapped at various points according to the amount of currents desired. The patient is placed in an induced current which arises from the solenoid, or if we may so regard it, in a shunt or branch of the circuit, the currents being conducted from this solenoid and applied by electrodes with insulating handles. In another method the patient is placed inside a huge solenoid built up on a wooden frame, or on an insulated couch, having a large metal plate inserted in it. One of the Leyden jars is connected with the plate and the other with the patient. Again, one end of the small solenoid may be connected with the earth, and the currents from the other may be led through an Oudin resonator and employed to give a brush discharge to the patient.

An interesting point is that one variety of these currents was really used by Morton, of New York, under the name of the induced static current, before their nature was understood. He employed a static machine, on the arms of which two small Leyden jars were hung, and he reduced the spark gap to almost nothing. The form of the currents has since been investigated and found to be an oscillating one, depending on the extremely rapid to and fro discharges across the spark gap. These oscillations may amount to hundreds of millions of cycles in a second. Jacoby compares them to the vibrations of a straight spring fixed at one end and suddenly released from a position of tension. In the same way a fluid contained in a U-tube, if disturbed, will oscillate up and down before it reaches a state of rest in both arms. The remarkable thing about these currents is that they can pass through the human body without causing pain or harm, though they may be obtained from currents of immense voltage and deadly character. Thus an alternating current from the main of 110 volts, if used as their source, may be raised in a Tesla transformer to several thousand volts, but a third change is effected in the condensers giving these rapidly oscillating currents of a harmless type. Hence, if the Tesla apparatus is used, most careful and complete insulation must be ensured for the currents until they are changed in the condensers. For reasons of safety it is desirable in medical practice to obtain the currents through the medium of a static machine or a spark coil instead of a Tesla apparatus, though even then care must be taken to get thorough insulation of the currents in the intermediate stage.

(*To be continued.*)

